# Long-Term Management and Monitoring of the Bladder After Spinal Cord Injury in a Rodent Model

**DOI:** 10.3390/biology14040373

**Published:** 2025-04-04

**Authors:** Michael Kleindorfer, Elena Esra Keller, Karin Roider, Evelyn Beyerer, Patrick Heimel, David Hercher, Martha Georgina Brandtner, Lukas Lusuardi, Ludwig Aigner, Sophina Bauer

**Affiliations:** 1 Institute of Molecular Regenerative Medicine, Paracelsus Medical University, 5020 Salzburg, Austria; 2 Department of Urology and Andrology, Landeskrankenhaus—University Clinic, Paracelsus Medical University, 5020 Salzburg, Austria; 3 Core Facility Hard Tissue and Biomaterial Research, Karl Donath Laboratory, University Clinic of Dentistry, Medical University Vienna, 1090 Wien, Austria; 4 Ludwig Boltzmann Institute for Traumatology, The Research Center in Cooperation with AUVA, 1200 Vienna, Austria; 5 Austrian Cluster for Tissue Regeneration, 1200 Vienna, Austria; 6 Department of Pediatric and Adolescent Surgery, Landeskrankenhaus—University Clinic, Paracelsus Medical University, 5020 Salzburg, Austria

**Keywords:** animal model, spinal cord injury (SCI), 3R, neurogenic lower urinary tract dysfunction (nLUTD), neurogenic bladder

## Abstract

Spinal cord injury (SCI) is a complex clinical condition with a wide range of permanent functional and neurological consequences. A prime factor limiting the patient’s quality of life (QoL) is difficulties in bladder function. Chronic animal models that help to develop novel therapeutic strategies are highly demanded, but their availability is scarce and frequently accompanied by substantial limitations. We want to provide our detailed protocols that allow full reproducibility of a novel model for investigating both the acute and chronic condition, and give transparency regarding challenges. The preclinical animal model of female rats with mid-thoracic SCI contusion and a permanently implanted urinary catheter allowed the measuring of bladder function repetitively. Over a period of six months, data were collected weekly from the same, conscious individuals. To our knowledge, this is the first study that obtained a clinically relevant urodynamic dataset seamlessly from the acute to the chronic phase in rats with SCI. The ability to generate a complete data set from one single individual, rather than requiring multiple subjects, has the potential to markedly reduce the number of experimental animals, eliminate group differences, and give more flexibility for therapeutic intervention. Future projects could also benefit from the described optimizations in animal care.

## Simple Summary

Spinal cord injury is an event that marks a turning point in the life of people worldwide. Often caused by traumatic accidents, injuries can lead to severe limitations of movement and sensation. Moreover, bladder dysfunctions are one of the most frequent daily struggles and an important factor for quality of life in patients. However, available treatments still do not fully meet the needs of these patients and cause strong side effects. Animal models are important for discovering new therapies, but their availability is scarce and often accompanied by substantial limitations. There is a high need for models generating a comprehensive and clinically relevant data set of bladder function after spinal cord injury. In this work, we describe a novel rat model, which for the first time enables health care providers to follow bladder function seamlessly from the acute to the chronic phase after spinal cord injury in the same individuals. This not only creates a versatile model for testing various therapeutic strategies, but also has the potential to markedly reduce the number of experimental animals needed in future projects. Together with described optimizations in practice and care, this could represent a valuable contribution to animal welfare.

## 1. Introduction

Spinal cord injury (SCI) is a devastating and life-changing event that affects more than 0.9 million persons every year [1]. As the central nervous system (CNS) has a very limited ability for recovery [2], injuries may lead to permanent loss of sensation, motion, and autonomic function [1,3]. One autonomic function, which is also a major factor in quality of life (QoL), is the loss of voluntary bladder control [4,5]. Depending on the lesion site, nearly all patients (94.9%) develop neurogenic lower urinary tract dysfunction (nLUTD) after SCI [6]. nLUTD may lead to high intravesical and sphincteric pressures and cause incontinence, urinary tract infection (UTI), and bladder stones. In the long term, nLUTD may even damage the upper urinary tract (UUT) and lead to kidney failure [7,8]. Current treatment strategies are mainly focused on symptomatic therapies to avoid UUT damage, but fail to fully address the underlying pathology. To find novel therapeutic solutions, animal models replicating frequently seen types of SCI are crucial. Among these models, the rat is the most commonly used one [9,10,11,12] because of its similar pathophysiology to humans and its accessibility to most research groups [13]. However, there is only a small number of studies that focus on the dynamic changes within the bladder after SCI, assessed by repetitive urodynamic measurements. Especially, data based on chronic conditions with a follow-up period (FUP) of more than three months are missing almost completely. According to a recent review [9] on nLUTD assessments in animal models with SCI, the majority of SCI rodents were followed up for several days up to a maximum of 2 months with terminal filling cystometries. Only one long-term rat study was listed with up to 4 months follow-up and a single final urethane-based filling cystometry [14]. Cats and dogs are used for nLUT SCI studies for a long time and allow longer follow-up monitoring. Yet, they share similar limitations as the use of anesthetics for filling cystometries and the lack of repetitive measurements in single animals [15,16,17]. Pigs or minipigs might be the most translational animal model routinely used for urological research, as they can be monitored for longer FUP after SCI with repetitive awake urodynamic measurements using the same urodynamic set up as in clinical routine [18]. However, this model is not suited for every research question, and the substantial requirements of both personal and financial resources are limiting factors for its implementation by other research groups. Regarding the use of more advanced repetitive awake urodynamics in rodents, we and others have implemented a novel technique to enable extended follow-up periods with permanently implanted urinary catheters to monitor bladder pathologies [19,20,21]. With our newly implemented flushing protocol, we aim to assure patency of the catheter for longer periods post-SCI. To our knowledge, we are the first to collect a data set of six months in rats with moderate to severe mid-thoracic spinal cord contusion. This allowed seamless monitoring of the recovery phase in a single individual from the acute until the chronic state, while bladder behavior was followed thoroughly, and potential group differences were eliminated. With this versatile model, therapeutic intervention can be tested at various time points after injury to identify the ideal time points for drug administration or optimize the efficacy of treatments. We want to provide a guideline for other research groups to assure full reproducibility of this model and to overcome the major challenges and complications. It is essential to communicate these aspects clearly and transparently, not only to secure the successful acquisition of high-value data for an extended period post-injury, but also to benefit animal welfare.

## 2. Materials and Methods

### 2.1. Experimental Animals

Three-month-old female strain Lewis rats (*n* = 12) from Charles River Laboratories (Sulzfeld, Germany) were used for the study. Standard housing conditions (12-h light/dark cycle, 40–60% humidity, 22 °C) and ad libitum access to food and water were provided. An amount of 3–4 S bedding material (Safe^®^, Rosenberg, Germany) consisting of fibrillated fibers was chosen because of its softness and liquid absorbance. Upon arrival, all animals underwent two weeks of acclimatization and handling before surgery in order to minimize stress and enable their adjustment to the new environment, food, and experimenters [22]. Ethical approval from the Austrian Governmental Legal Entity on Animal Research was obtained (BMBWF-66.019/0030-V/3b/2019). All work was conducted in accordance with the local guidelines on animal use and the common ARRIVE guidelines.

### 2.2. Permanent Bladder Catheter Implantation

A bladder catheter was made of polyethylene tubing, with an inner diameter of 0.58 mm and outer diameter of 0.965 mm (PE-50). To minimize the risk of undesired catheter movement, the catheter was modified before the surgery. The last two millimeters of the catheter, which were in direct contact with the bladder wall, were covered with a silicone tubing of slightly larger size to increase friction. Moreover, the end of each catheter, which reached into the bladder cavity, was flared to prevent it from being pulled out of the bladder. All catheters were tested for their patency before the surgery. Under general anesthesia, using a combination of medetomidine (0.15 mg/kg), midazolam (0.08 mg/kg), and fentanyl (0.01 mg/kg) injected intramuscularly into the thigh, the catheter was implanted. By performing a low mid-line laparotomy at the level of the third and fourth teat, the bladder was exposed. Through a small incision in the bladder dome, the catheter was inserted into the bladder and fixed at this position by a 6-0 non-absorbable monofilament suture. After passing through the abdominal wall, the catheter was guided subcutaneously to the neck, exteriorized at the level of the scapula, and fixed to an infusion harness. With the infusion harness worn, the urinary catheter of each rat was available for the researcher at any time, but inaccessible for the animal’s manipulation. The screw-off lid allowed easy access for repetitive urodynamic measurements, but at the same time provided a reliable sealing. For a more detailed description and visual demonstration, please refer to our publication in the journal of visualized experiments [21]. As the rats remained in the harnesses throughout the experiment, cages were equipped with a transparent acrylic glass board, which kept the individual animals separate to prevent harness removal, but still allowed some visual interaction and olfactory sensing. For analgesic and antibiotic coverage, meloxicam (2 mg/kg, twice daily, s.c.) and enrofloxacin (5 mg/kg, once daily, s.c.) were administered for the first five days post-surgery. The rats were checked at least twice daily for signs of pain according to the grimace scale [23].

### 2.3. Spinal Cord Contusion

Two weeks after bladder catheter implantation, the SCI surgery was conducted. Under the same above-mentioned general anesthesia, a standard laminectomy was performed at the thoracic vertebrae Th8/Th9 and the spinal cord impacted with 250 kilodyne of force with an Infinite Horizon (IH) impactor [24,25]. Meloxicam (2 mg/kg, daily, s.c.) and enrofloxacin (5 mg/kg, daily, s.c.) were administered during the first five days post-surgery, together with Nutri-Cal (500 µL, daily, oral) as a food supplement. Montelukast (10 mg/kg body weight, daily, oral) was given for anti-inflammatory purposes, and additional enrofloxacin (5 mg/kg, daily, s.c.) upon requirement to counteract persistent UTI during the FUP. All animals were checked daily for sufficient water intake and signs of dehydration. Subcutaneous injections of saline solution were given in case of suspected dehydration. After SCI, the bladders were emptied manually twice daily to lower the risk of UTIs by evacuation of residual urine and prevent bladder rupture caused by overfilling. Urine volumes, discoloration and clarity were noted in a bladder diary. The rats were checked at least twice daily for signs of pain according to the grimace scale [23].

### 2.4. Catheter Flushing Protocol

To ensure the patency of the implanted urinary catheter for an extended period after SCI and prevent clogging caused by accumulated minerals or the formation of a biofilm, we designed a tailored flushing protocol. After manually emptying the bladder, 1 mL of sterile 3.23% citric acid solution was infused into the catheter and incubated for 5 min. The catheter was then flushed with 1 mL saline solution and the bladder expressed manually again. This was done twice a week as a standard procedure, starting one week after SCI. In cases of blockage, it was repeated for three consecutive days. If the catheter was still not fully patent, we performed an additional flushing with 0.02% polyhexanide solution (Uro-Tainer, Braun^®^, Melsungen, Germany) instead of citric acid.

### 2.5. Animal Welfare

Long FUPs after SCI with a permanently implanted catheter pose a number of challenges for the well-being of experimental rats. Besides the increased general risk of developing lower UTIs that may arise from a dysfunctional bladder after SCI, the urinary catheter itself may be the cause of a systemic infection, leading to sepsis and death. In the case of suspected UTI, a urine strip test was performed to confirm an elevated neutrophil count before enrofloxacin was administered. Besides the 3–4 S bedding material (Safe^®^) and a sufficient amount of Sizzle-Nest (datasand^®^, Manchester, UK) nesting material, cages were additionally equipped with different enrichments tailored to the needs and abilities of the injured rats and their worn infusion harnesses. Accessibility to supplied food and water was assured at all times. Shortly after the SCI injury, food was also placed directly at ground level for the animals. In case of overly wet bedding or nesting material, it was partly exchanged. Close monitoring of the body weight and keeping of a bladder diary helped to identify early signs of inflammation. To avoid abrasion wounds under the silicone straps of the infusion harnesses that had to be fixed tightly to the rats, antibiotic Baneocin powder (bacitracin zinc 250 IU, neomycin sulfate 5000 IU (5 mg/g)) was used if needed. Risk of decubitus formation due to the constantly wet and irritated skin caused by spilled urine was lowered by regular washings and creaming with Pantothen upon requirement. In case of progressive decubitus ulcers, Cavilon and Cavilon Advanced were applied to provided a stretchable and water-repellent protective coating for the affected skin [26,27].

### 2.6. Locomotion Scoring

Two experimenters scored their ability to use their hind limbs on the 21-point BBB locomotion scale [28]. Rat joint movement, paw placement, weight support, stepping, fore limb–hind limb coordination, trunk stability, and tail position were analyzed at baseline and after SCI at 1, 8, 28, 45, 53, 91, 119, 147, and 172 days post-injury (DPI).

### 2.7. Awake Cystometry

Shortly before starting the weekly cystometric recording at the Catamount cystometry station (CCS) [21], the bladders of the awake rats were manually expressed. Animals were placed in a restrainer (Kent Scientific^®^, Torrington, CT, USA) that limits movement but allows accessing the lid on the infusion harness. The animal position was in a way that the bladder was at the exact same height as the pressure transducer. A hole in the bottom of the restrainer at the position of the animal’s urogenital area allowed the urine to freely fall onto the scale (ML303E, Mettler Toledo^®^, Vienna, Austria) that was positioned underneath. After calibration of the cystometry unit, the end of the rat’s implanted urinary catheter was connected to the system and saline solution was infused (R100-EC Syringe Pump, Razel Scientific Instruments^®^, Saint Albans, VT, USA) into the bladder at a constant rate of 120 µL/min until a minimum of three micturition cycles were measured. When no micturition occurred, the filling was stopped before the estimated maximum bladder capacity, based on the highest residual volumes of the last week, was reached. During the recording, the rats were monitored closely and any events such as strong movements, fecal excretion, or spasms were noted. To rate bladder function, a scoring system based on patterns was developed together with an experienced neuro-urologist. Thus, we were able to compare and visualize bladder recovery. A maximum score of four points was assigned to healthy and normal bladder behavior, while a score of three described the presence of an active voiding reflex in presence of non-voiding contractions (NVC). At a score of two, only urine leakage could be observed, but the detrusor was still active. One score point was the lowest rating and denoted a fully dysfunctional bladder with only urine leakage but no measurable detrusor activity. Primary scoring of the acquired data was performed by the researchers, who selected a representative of 30-min duration within each measurement, subtracted all noted external events, and assigned it to the respective score. This process was then validated by two neuro-urologists in a second step.

### 2.8. µCT of Spinal Cord

In deep anesthesia, by injected a mixture of ketamine (546 mg/kg), xylazine (14.2 mg/kg) and acepromazine (1.3 mg/kg) intraperitoneally, animals were flushed blood-free with phosphate-buffered saline (PBS) supplemented with 10 U/mL heparin via the heart, and then perfused with 4% paraformaldehyde (PFA). Spinal cords were harvested, post-fixed for 1 h in 4% PFA and then stored in PBS until further processing. Tissue was later incubated in Accupaque-350 (GE Healthcare, Munich, Germany) diluted 1:2 in PBS for 48 h. Scans were performed in a SCANCO µCT 50 at 70 kVp with 85 μA and a 0.5 mm Al Filter. A total of 3400 projections/180° were integrated 4 times for 650 ms and averaged. The scans were reconstructed to an isotropic resolution of 6 μm. Approximately 21 mm of spinal length was later scanned with the lesion epicenter positioned in the center of the scan. The scans were evaluated using Fiji (ImageJ v1.53a) [29] according to a procedure described by Romanelli et al. [30]. Spinal cords were aligned according to the lesion epicenter and the average cross-section area of spinal cord, damaged tissue, and cyst was measured for each 60 µm segment along the spinal cord. Damage regions were recognized by textural change or an increase in signal intensity.

## 3. Results

### 3.1. Confirmation of Spinal Cord Contusion

**Figure 1:**
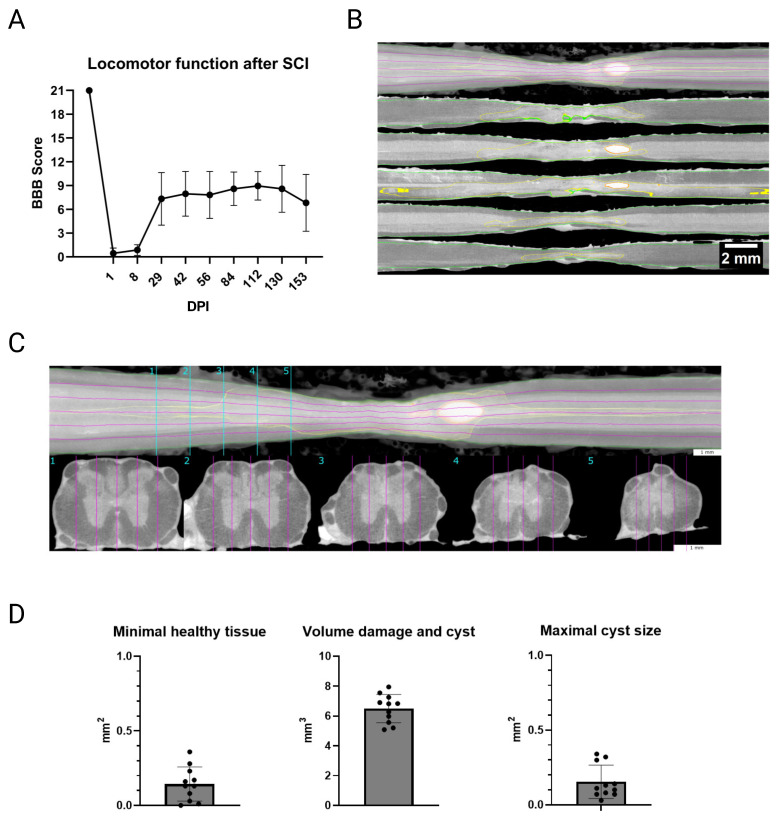
(**A**) After contusion spinal cord injury (SCI), the rats experienced a comparable drop-down of locomotion on the BBB to a maximum score of one (mean of both hind limbs) one day post-injury (DPI). A plateau was observed at a maximum score of 11 at 29 DPI. The rats failed to achieve better functionality during the six-month follow-up period. Values shown are means ± standard deviation. (**B**) µCT scans of the spinal cords were used to visualize and quantify the damage to the spinal cord at the level of injury at Th8/Th9. Top: Volume rendering of the spinal cord. Bottom: Five curved longitudinal slices that were equally distributed relative to the width of the spinal cord to follow the actual geometry of the strongly distorted, injured spinal cord. The magenta lines in the volume rendering indicate the slice position of the single curved longitudinal slices. They are equally spaced along the width of the spinal cord. The clear healthy regions are indicated by green margins, injured tissue by yellow margins, and cyst formations represented in red. Scale bar: 2 mm. (**C**) Volume rendering of the spinal cord with five representative cross-sections at different locations relative to the lesion site. Scale bar: 1 mm. (**D**) Quantified values from the µCT images of the thoracic spinal cord revealed consistent and severe defects in all rats. Values are shown as means ± standard deviation.

Feedback on the quality of the injury was given by the BBB locomotion score, which confirmed a complete drop-down to paraplegia, represented by a maximum score of 1 point (mean of both hind limbs) at 1 DPI and 2 points at 8 DPI (Figure 1A). A plateau at a maximum score of 11 was reached at 29 DPI, which is described as frequent to consistent weight-supported plantar steps, but no front-limb to hind-limb coordination. Retrieval of coordinated walking was not observed by any rat. However, there was some variation between the animals and their individual recovery. Additionally, µCT scans clearly showed consistent defects and minor cyst formations in all rats (Figure 1B,C). Minimal healthy tissue fell to a mere 0.14 mm^2^, while the volume of damage on comparison was 6.49 mm^3^. The maximal cyst size was on the average about 0.15 mm^2^ (Figure 1D). Accordingly, the SCI contusion resulted in comparable functional deficits and tissue damage in all rats.

### 3.2. General Animal Welfare

**Figure 2:**
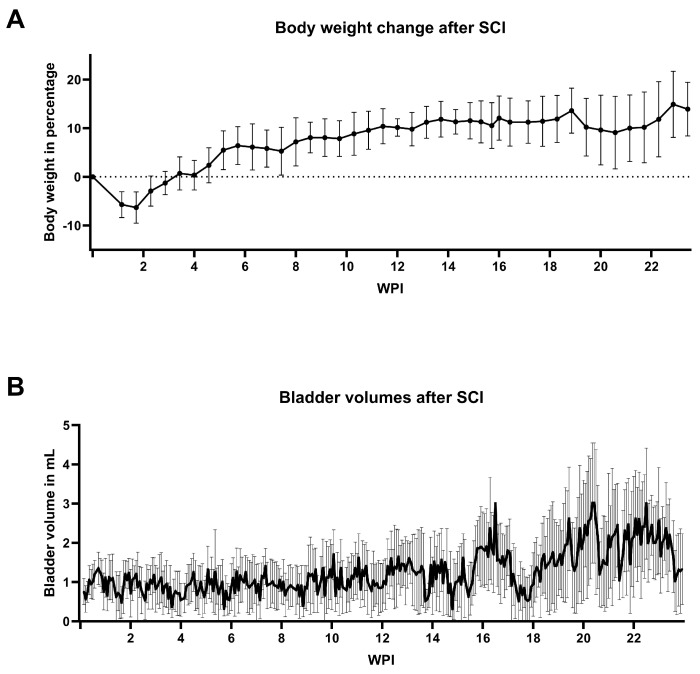
(**A**) After contusion spinal cord injury (SCI), the rats lost about 10% of their body weight within the first two weeks post-injury (WPI), which they regained in two weeks’ time. Values shown are means ± standard deviation. (**B**) Bladder volumes did increase after SCI over the course of 24 WPI. From an average of about 1 mL shortly after the SCI, a volume in excess of 2 mL was noted during the second half of the follow-up period. Values shown are means ± standard deviation.

Eleven rats reached the 6-month FUP; only one rat was lost during the early postoperative phase. Experienced weight loss of about 10% within the first two weeks post-SCI was regained after a further two weeks. Overall, there was a steady gain in body weight observable for the entire duration of the experiment, which was only interrupted by shorter periods of stagnation or slight decrease (Figure 2A). One rat needed extended skin care beyond the usual washing procedure. Due to marked irritation, the rat first received Pantothen cream on three consecutive days, followed by six Cavilon applications over a period of twelve days. As this treatment did not yield the desired result, Cavilon Advanced was applied ten times over the next 35 days. Cavilon Advanced was able to improve the rat’s condition, reverse the progressing decubitus ulcer, and restore intact skin. One additional animal had to be excluded from all urodynamic measurements because its urinary catheter had to be explanted at 51 DPI, after the animal showed signs of severe inflammation with potential risk of sepsis and endocarditis. While the reimplantation of a new catheter at 79 DPI was successful, it did not stay inside the bladder and detached after a few days.

### 3.3. Bladder Welfare

**Figure 3:**
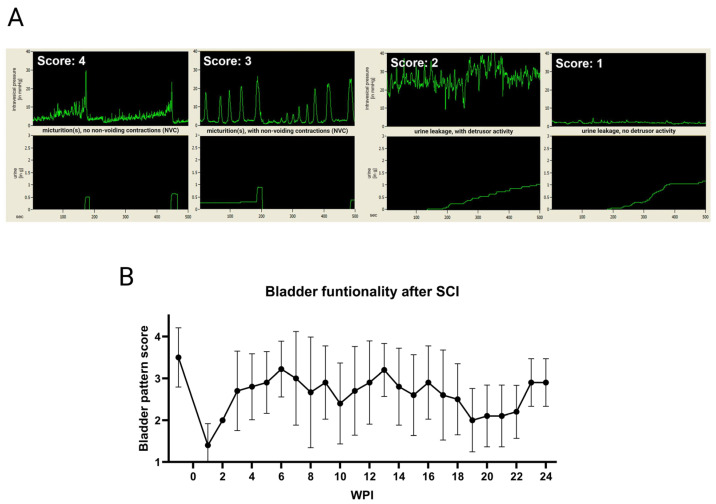
(**A**) Weekly urodynamic measurements in the conscious animals were scored based on patterns that resembled specific bladder functionality after contusion spinal cord injury (SCI). During continuous filling of the bladder at a rate of 120 µL/min, intravesical pressure (in mmHg) and voided urine (in grams) were recorded and displayed against time (in seconds). The highest score of four denoted normal micturition and the absence of non-voiding contractions (NVC), whereas a score of three was given in the presence of NVC. For visual purposes, the scale collecting the voided urine was reset to zero after each micturition. When only urine leakage was observable, but the detrusor was still active, this was assigned to a score of two. The lowest score of one denoted the least functionality and only urine leakage, with no visible detrusor activity. Shown measurements are representative and were not recorded in the same individual. (**B**) The rats showed a drastic drop-down in functionality during the first two weeks post-injury (WPI), but regained some functionality after about three weeks. Values shown are means ± standard deviation.

Mean bladder volumes of the manual expression increased during the FUP from an average of about 1 mL in the first three months to values above 2 mL (Figure 2B). Antibiotics were administered to all rats due to UTI for a duration of four to five days at 12, 15, and 21 weeks post-injury (WPI). Some individual rats received additional treatment at three other time points. All implanted urinary catheters remained functional and patent for the entire six-month FUP, with the exception of one individual, which needed explantation as described above. Slight clogging was observed 29 times in total, but reopening with gentle pressure via a connected syringe during the regular flushing with citric acid was sufficient to resolve the condition in all cases. Only once, additional polihexanide flushing was needed to ensure a completely patent catheter lumen. The provided protocol for following up a clogged catheter was used thereafter. Urodynamics were successfully measured on a weekly basis over the entire 6-month FUP. The time of recording usually ranged between 40 and 80 min, always adjusted to the number of micturitions, but also considering the estimated maximum bladder volume according to the bladder diary. Bladder patterns showed a drastic drop-down of functionality within the first two WPI, while some functionality was regained after three WPI (Figure 3). Between 19 and 22 WPI, a slight drop in the bladder functionality score is apparent; however, the score increased again at 23 WPI. Normal bladder function was not re-achieved by the rats after the SCI.

## 4. Discussion

The main aim of this study was to create a versatile SCI animal model to consistently collect clinically relevant data on specific bladder pathology from the early stages after injury to the chronic state. A key element of this was a customized long-term care strategy for the rats and their implanted urinary catheters.

In total, we aimed to collect data on twelve animals over a period of six months. While one rat was lost in the early postoperative phase, a second individual required explantation of the urinary catheter due to signs of severe inflammation. As the reimplanted catheter did not stay in the bladder, the rat had to be excluded from further urodynamic testing. The animal loss is in line with our previous experience and the existing published data [31,32,33]. However, information about exact dropout rates were scarce in the literature. With ten animals completing the six-month FUP and all functional assessments of the bladder, to our knowledge, we are the first to collect such a comprehensive data set from rats. This allows for fully covering all states of the pathology and tracking the dynamic changes of the bladder after injury in one individual, which would otherwise require the use of several rats. We expect this to markedly reduce the number of experimental animals needed in many future studies, while providing much more flexibility to investigate various aspects of bladder function and improving animal welfare. The provided detailed protocols should assure the reproducibility of this model and serve as a guideline for overcoming the major challenges.

To maintain general health and counteract the anticipated drastic loss in body weight after SCI, we supplied the rats with an additional nutritional intake using Nutri-Cal [34] for the first five DPI. However, supplementation might not have been a crucial aspect in our project; unexpected weight loss of single individuals is a considerable risk that can lead to exclusion. Another important routine was regular washing of the animals to prevent skin irritation due to spilled urine. To curtail decubitus ulcers, Pantothen cream and Cavilon spray failed to achieve the desired skin protection and healing, while Cavilon Advanced was able to improve the rats’ condition and ultimately resolve the wound. In addition to softer bedding material and other wound treatment options, Cavilon Advanced may well be included in the repertoire of decubitus prevention. With the rats in good health and the catheter lumen kept open, the awake urodynamic measurements could be performed weekly for six months. This is a substantial improvement, as the catheter is particularly prone to becoming clogged due to the accumulation of minerals or the formation of a biofilm, which typically makes the bladder inaccessible approximately three months after catheter implantation [21]. The extensive data were analyzed by scoring actual bladder functionality based on patterns we developed in cooperation with an experienced urologist. This allowed for the immediate identification of reactions and changes in bladder function, similar to clinical routines. Immediately after SCI, bladder dysfunction became very apparent, and recovery occurred only to a certain extent. When conditions progress to chronic, a multitude of factors dictate bladder function or provoke dysfunction. Despite being the basis for urodynamic data acquisition and representing one of the widely adopted standard methods in the field, the implanted urinary catheter itself can also be seen to contribute to these factors. Because of its rigid structure or the potential to provoke an inflammatory response, effects on normal bladder behavior cannot be fully ruled out. However, with the repetitive measurements, the sum of all these influential effects might be compensated better.

Another aspect that was crucial for recreating this specific type of bladder dysfunction was the properties of the preceding SCI. Therefore, we selected the position and contusion force accordingly. To confirm the severity of SCI, the BBB locomotor score was the method of choice, as it was originally designed for scoring of rats with thoracic SCI [28]. After the initial drop to paraplegia, some functionality was regained within the first 29 DPI, reaching a maximum score of eleven. This was expected and reflects the severity grade post-SCI [35,36]. The observed variations in scoring after the spinal shock phase may be explained by the well-known limitations of the BBB score, such as the rough scaling or ceiling effects [37]. Compromised general health or an underlying UTI—aside from the necessity to house the animals separately [37] and the constantly worn infusion harness—might also affect their performance. The options for assessing locomotor function differently were limited, as most tests require the ability of frequent to consistent weight-supported steps for a reasonable application [35,38]. However, those that do not involve gait or stepping pose a potential health risk for the animals and their implanted urinary catheters, or are prone to being affected by adaption to the test [37,39]. Further characterization of the injury site through ex vivo µCT analysis of the spinal cord also confirmed almost identical tissue damage at the correct vertebral level for all rats. Although a transection would yield the most consistent and reproducible SCI, we have selected a contusion model as it mimics the human condition more closely [1,3]. Based on our previous experience with comparable rat models, which includes execution of the injury hit by the IH impactor, we could keep intraindividual variations of the SCI at a minimum. Nevertheless, individual reaction and recovery of the animals to some extent always needs to be considered.

## 5. Conclusions

The combination of an SCI animal model with an implanted urinary catheter for repetitive measurements and a customized flushing protocol created a versatile model for therapeutic applications. Bladder function could be monitored for the first time through all stages of the pathology after SCI in a single rat, which would otherwise require the use of several individuals. With additional improvements in practice and care, this can lead to a markedly reduced number of experimental animals and improved animal welfare. We expect all these aspects to be valuable contributions to the 3R principle.

## Data Availability

The data associated with the paper are not publicly available but are available from the corresponding author on reasonable request.

## Abbreviations

The following abbreviations are used in this manuscript:

BBB	Basso, Beattie, and Bresnahan
CCS	Catamount cystometry station
CNS	Central nervous system
DPI	Days post-injury
FUP	Follow-up period
IH	Infinite horizon
nLUTD	Neurogenic lower urinary tract dysfunction
NVC	Non-voiding contractions
PBS	Phosphate-buffered saline
PFA	Paraformaldehyde
QoL	Quality of life
SCI	Spinal cord injury
UTI	Urinary tract infection
UUT	Upper urinary tract
WPI	Weeks post-injury
